# Biomechanical Evaluation of a Tooth Restored with High Performance Polymer PEKK Post-Core System: A 3D Finite Element Analysis

**DOI:** 10.1155/2017/1373127

**Published:** 2017-03-12

**Authors:** Ki-Sun Lee, Joo-Hee Shin, Jong-Eun Kim, Jee-Hwan Kim, Won-Chang Lee, Sang-Wan Shin, Jeong-Yol Lee

**Affiliations:** ^1^Department of Prosthodontics, Korea University Guro Hospital, Seoul, Republic of Korea; ^2^Department of Conservative Dentistry, Korea University Guro Hospital, Seoul, Republic of Korea; ^3^Department of Prosthodontics, College of Dentistry, Yonsei University, Seoul, Republic of Korea; ^4^Graduate School of Clinical Dentistry, Korea University, Seoul, Republic of Korea

## Abstract

The aim of this study was to evaluate the biomechanical behavior and long-term safety of high performance polymer PEKK as an intraradicular dental post-core material through comparative finite element analysis (FEA) with other conventional post-core materials. A 3D FEA model of a maxillary central incisor was constructed. A cyclic loading force of 50 N was applied at an angle of 45° to the longitudinal axis of the tooth at the palatal surface of the crown. For comparison with traditionally used post-core materials, three materials (gold, fiberglass, and PEKK) were simulated to determine their post-core properties. PEKK, with a lower elastic modulus than root dentin, showed comparably high failure resistance and a more favorable stress distribution than conventional post-core material. However, the PEKK post-core system showed a higher probability of debonding and crown failure under long-term cyclic loading than the metal or fiberglass post-core systems.

## 1. Introduction

Intraradicular dental post-core systems have been widely used for the restoration of teeth that have lost a considerable amount of their crown structure [1, 2]. The most widely used post-core systems can be classified into two basic types: one-piece cast post-core system and two elements system comprising a prefabricated post with composite core. Traditionally, metal alloy post-core systems are preferably chosen for the restoration of the tooth in such status, because they are easily customized to the various shapes of the root canal and have superior mechanical strength [1, 3]. However, due to a large elastic modulus difference between metal alloys and dentine, an excessive functional stress concentration may occur around the post, resulting in catastrophic root fracture [1, 2, 4]. Therefore, to achieve long-term safety, various post-core materials have been investigated. According to previous studies, when using a lower elastic modulus post material, such as fiberglass, a more favorable stress distribution occurs [5–12]. However, since fiberglass posts are generally supplied as ready-made products, they are limited in terms of their conformity to the shape of the root canal. In addition, although fiberglass posts have lower elastic moduli (from 45.7 to 53.8 GPa [13]) than those of metal alloy posts (110.0 GPa for titanium and 95.0 GPa for gold [14]), these are still approximately three times the elastic modulus of dentin (18.6 GPa [15]).

Recently, biocompatible high performance polymers, PolyEtherKetoneKetones (PEKKs), were introduced as novel dental materials. Due to their acceptable fracture resistance, better stress distribution, and shock-absorbing ability, high performance polymers are considered as alternative dental materials for metal and glass ceramics [16]. PEKK is one of the organic thermoplastic polymers in the PolyArylEtherKetone (PAEK) family, best-known as a high performance polymer family, and mainly serves as an implantation material due to its aforementioned features and good biocompatibility in the medical field [17]. It has been recognized as an adequate alternative biocompatible material for long-term proven titanium in orthopedic applications [18–20]. In the dental area, the main usage of the PAEK family has increasingly been as temporary implant abutments [19–21]. In addition, it is used as dental clasps and frameworks for removable dental partial prostheses [22]. The manufacturer (Cendres+Métaux, Milano, Italia) reports that PEKK has a similar compressive strength (246 MPa) to that of dentine (297 MPa [14]), although it has a lower elastic modulus (5.1 GPa) than that of dentin. In addition to its biocompatibility, appropriate mechanical strength, shock-absorbing ability, and a wide capability of fabrication processing including milling and pressing make PEKK an attractive dental material for the fabrication of custom-made intraradicular dental post-core systems. According to the previous studies [17], PEKK can also use resin bonding systems when using the appropriate combination of mechanical surface treatments and primers. However, there have been no studies of this novel high performance polymer PEKK as post-core material. Therefore, the aim of this study was to evaluate the biomechanical behavior and long-term safety of PEKK as an intraradicular dental post-core material by comparing it with the other conventional post-core materials using finite element analysis (FEA).

## 2. Materials and Methods

### 2.1. Finite Element Model

This study was performed under approval of the Institutional Review Board (IRB no. MD13022) of Korea University Medical Center (KUMC). A randomly collected maxillary central incisor from the human tooth bank at the KUMC dental center was used to build the model. The geometry of the tooth was obtained using an Identica Blue® 3D dental scanner (Medit Inc., Seongbuk-gu, Seoul, Korea) and the 3D modelling software, Geomagic Wrap® (3D Systems Inc., Cary, North Carolina, United States). The geometry of the tooth-supporting bone was constructed with reference to a previous study [23].

**Figure 1:**
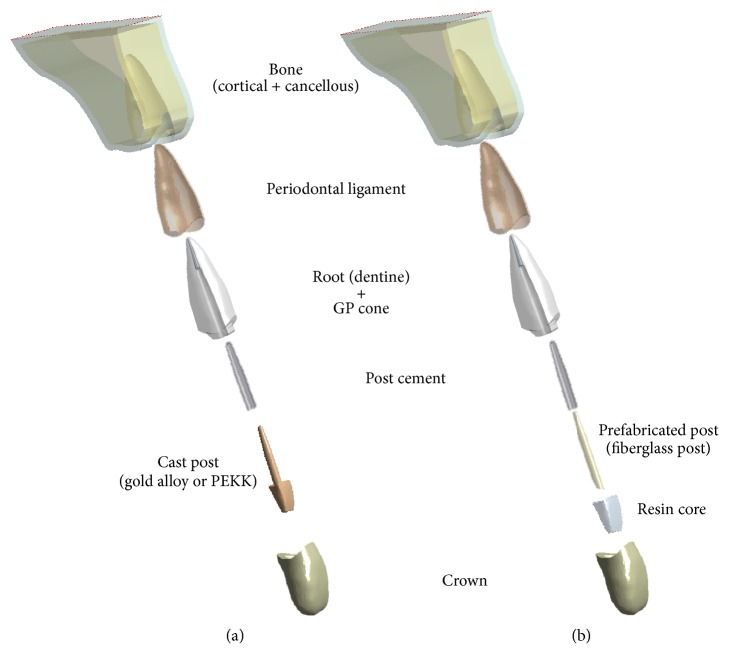
Components of constructed geometry models. (a) Post and core integrated model for simulating gold alloy post-core system and PEKK post-core system. (b) Post and core separated model for simulating prefabricated fiberglass post and resin core.

In this study, three different post-core systems were compared using the two types of geometry models, as shown in Figure 1. One of the geometry models was a post and core separated model to represent a prefabricated fiberglass post and a resin core system. The other geometry model was a post and core integrated model to represent a gold alloy post-core system and a PEKK post-core system. To evaluate only the influence of the post-core materials, excluding the influence of shapes, the dimensions of all post-core systems in this study were assumed to be the same. The reference model of the post in this study was Snowlight® (Carbotech, Ganges, France), because its tapered geometry is similar to that of the root canal of teeth. Following the general recommendation for post length and diameters [24], the post length was approximately three-quarters of root length and the post diameter obeyed the thickness ratio (post diameter/root thickness) = 0.2. The rest of the apical root canal space, apart from the space occupied by the post, was assumed to be filled with a Gutta-percha (GP) cone, and the final crown restorative material was assumed to be a dental ceramic. Finally, the components of a constructed geometry model consist of a maxillary bone, periodontal ligament, tooth root, GP Cone, cast post-core (or prefabricated post and resin core), post cement, and dental ceramic crown.

### 2.2. Stress Analysis

**Figure 2:**
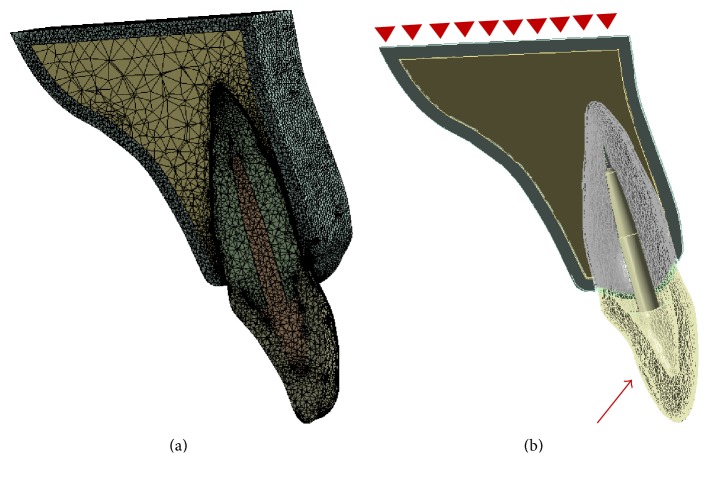
(a) Sagittal sections view of the finite element mesh of assembled post-core restored tooth model. (b) Sagittal section views of the boundary and loading conditions of the geometry model.

After the construction of the models, the designed models were transferred to the FEA software ANSYS 17® (ANSYS Inc., Canonsburg, Pennsylvania, United States). All models were then meshed using tetrahedral elements, as Figure 2 shows. The mechanical properties of the elements, assumed to be homogeneous, isotropic, and linear elastic in this model, were obtained from previous studies, as described in Table 1 [13, 15, 25]. The bottom faces of the bone models were fixed. Applied load to the models was directed at an angle of 45° to the longitudinal axis of the tooth and it was evenly distributed along the palatal surface. The stress induced in each component of experimental models was calculated as Von Mises Equivalent (VME) stresses, and stress distributions in the root were also assessed.

### 2.3. Fatigue Analysis

Fatigue can be defined as the progressive and localized structural damage that occurs when a material is under long-term cyclic loading [26]. The failure value of a material is generally lower than the yield strength of the material. For a fatigue analysis in the FEA study, *S*-*N* (stress versus number of cycles to failure) curves of the materials are required. The *S*-*N* curve of dentine was referenced from a previous study [27]. Generally, the *S*-*N* curves of materials, when no experimental data are available, can be estimated by the following formula (flexural strength (*S*_*f*_) versus number of cycles): (0.9 · *S*_*f*_ versus 10^3^), (0.5 · *S*_*f*_ versus 10^6^), and (0.5 · *S*_*f*_ versus a number of cycles greater than those tested) [25]. In this study, 1.2 million cycles are applied, and *S*_*f*_ was considered, as shown in Table 2 [15, 25, 28]. A cyclic load from 0 to 50 N with 1.2 million cycles is believed to simulate approximately five years of clinical service [27], and this protocol has been used in several fatigue studies [25, 29, 30]. Following a previous fatigue analysis for natural tooth, the Goodman fatigue equation model was applied in the FEA software [25].

After the fatigue analysis was performed, the Factor of Safety (FOS) of each component in the finite element model was measured to compare the influence of post-core materials to the long-term safety of the overall components in the simulated models. FOS indicates the ratio of the material strength to the subjected maximum stress in that material under cyclic loading. Under cyclic loading conditions, a smaller value of FOS means that the material is more prone to failure, and a value of 1 or less means that the material is in the plastic state [25]. The higher the FOS value, the higher the long-term safety, and the lower the FOS value, the lower the long-term stability under functional loading conditions.

## 3. Results

### 3.1. Stress Distribution

**Figure 3:**
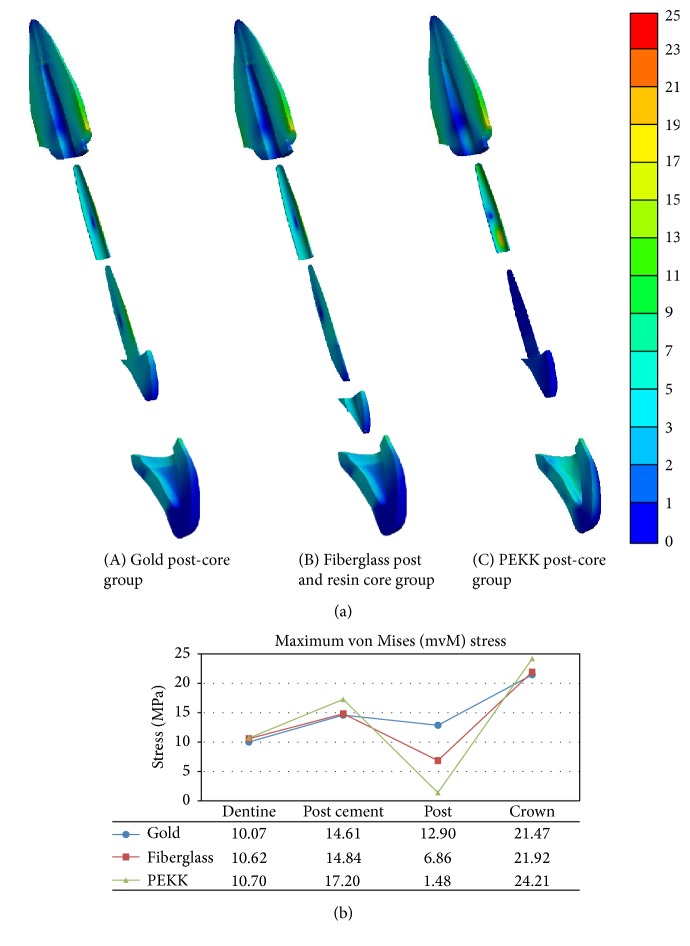
Sagittal section views for stress distribution of components in the study models (a). Maximum von Misses stress values of each component in the study models (b).

Figure 3 depicts the stress distribution in terms of color contour patterns and the maximum value of VME stress of each component. As shown in the figures, the gold and fiberglass post models demonstrated similar patterns and values of maximum equivalent stresses in the overall components of the model, except for the post. On the other hand, the PEKK post-core model showed more stress concentration at the crown and at the cervical area of post cement. However, it showed significantly less stress concentration in the post-core component than the other experimental models.

**Figure 4:**
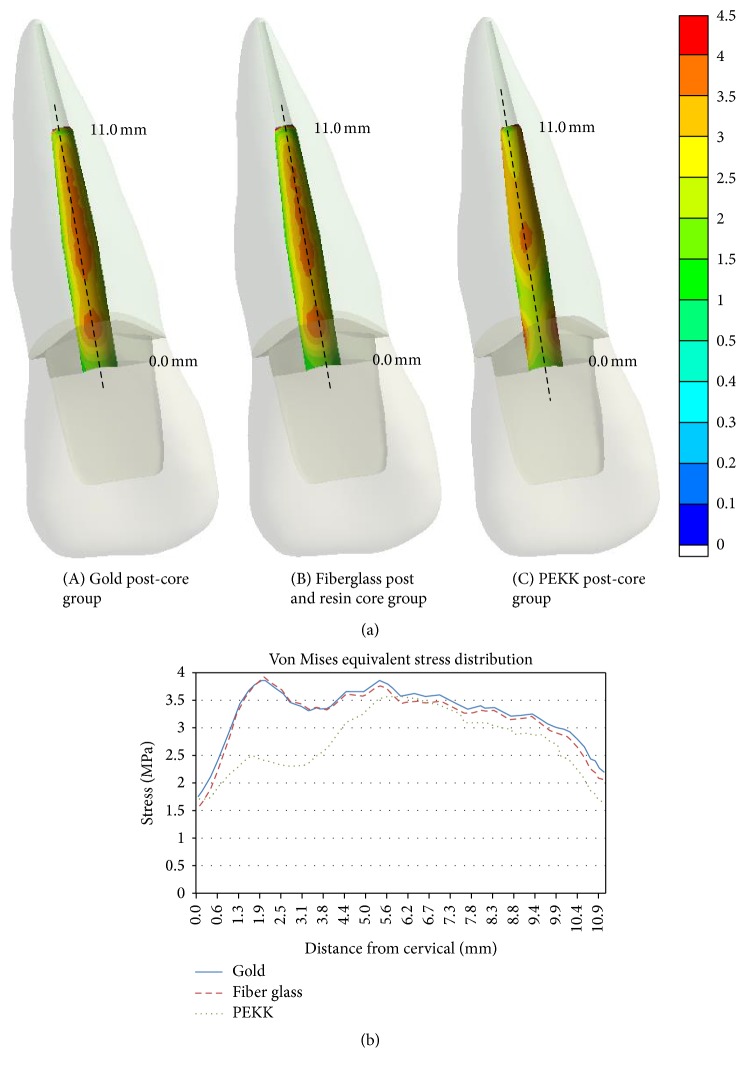
Coronal section views of VME stress distribution profile at the labial side interface surface of dentine and post cement along the root mid-plane. Distances are measured from the cervical region of the post to its apex.

Stress distribution profiles at the interface of dentine to cemented post along the root mid-plane in the three post-core restored models are shown in Figure 4. Distances in the figure were measured from the cervical region of a post to its apex along the longitudinal axis at the root mid-plane. In both the metal and fiberglass post cases, the maximum values of stress were located at cervical region and 1/2 of the root. In the PEKK post-core case, the stress distribution profile was lower than that of metal and fiberglass, and the maximum values of stress were located at 1/2 of the root only. In the cervical and apex region of the PEKK post-core case, the values of stresses were lower than those of metal and fiberglass cases. In addition, unlike the other cases of rigid post-core systems, the stress in the cervical region of PEKK was dispersed in the mesiodistal direction.

### 3.2. FOS of Fatigue Analysis

**Figure 5:**
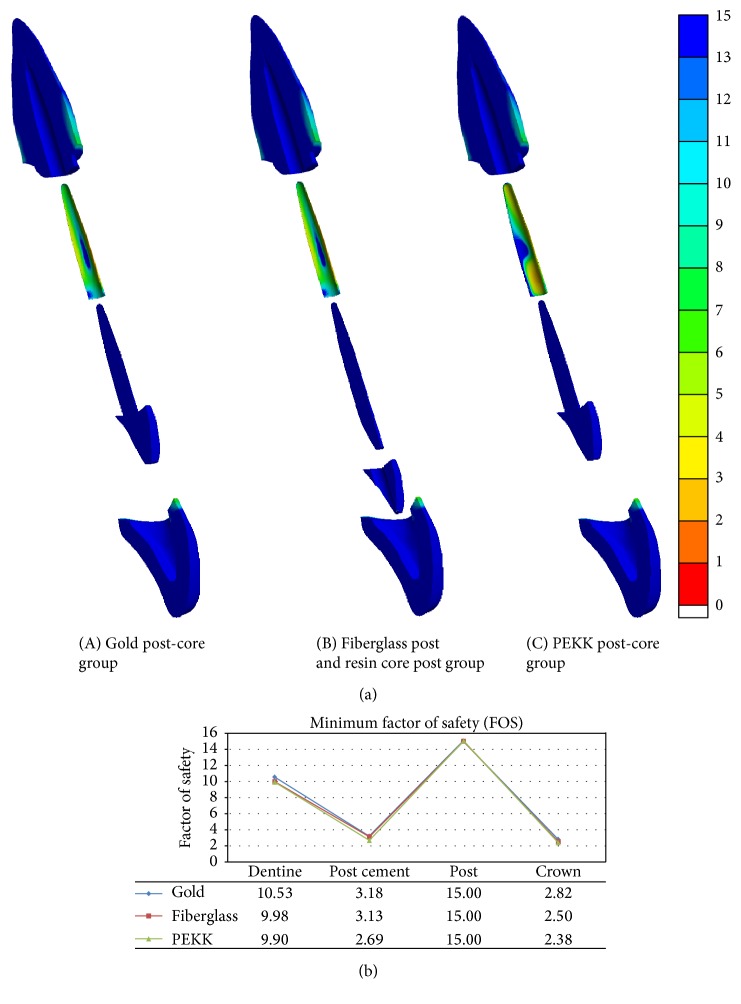
Sagittal section views of FOS distribution of each component in the study models (a). Minimum FOS of each component in the study models (b).

Figure 5 depicts the FOS distribution in terms of color contour patterns and the minimum value of FOS on each component. Regardless of the post-core system, all post-core components have the highest value of the safety factor, and all post cements and crowns showed the lowest values of approximately 3. However, in the tooth restored with PEKK post-core material, the extension of the smallest value of the FOS zone was larger than for other post-core models, and the minimum FOS value of post cement in the PEKK group was also slightly lower than for other models. The labial side FOS value of post cement in the PEKK group was higher than for the other groups.

## 4. Discussion

This study evaluated the use of the high performance polymer PEKK as an intraradicular post-core material by comparing it with conventional post-core materials. For a post-core-restored endodontically treated tooth, a root fracture is an undesirable incident. According to previous studies [31–33], one of the causes of root fracture of post-restored teeth is stress concentration around the post-apex. Clinically, when a high elastic modulus metal post and core is used as an intraradicular post and core in endodontically treated teeth, vertical root fractures often occur, which then lead to extraction of the teeth [31]. To prevent catastrophic vertical root fracture, a prefabricated fiberglass post and resin core is currently being used as a post and core system. Since fiberglass has a lower elastic modulus than metal but similar strength (Tables 1 and 2), fiberglass post systems induce favorable stress distributions within the root and generally exhibit a repairable horizontal fracture mode when root fracture occurs [5, 31]. However, while fiberglass has a lower elastic modulus than metal, its elastic modulus is still several times higher than that of dentine. Recently, the high performance polymer PEKK with an elastic modulus lower than that of fiberglass and similar to that of dentine has been introduced as an alternative intraradicular post-core material. However, there have been no experimental and clinical studies on the use of the material as a post-core system. Therefore, the present study was performed to evaluate the biomechanical behavior and long-term safety of PEKK post-core system in the radicular tooth structure by using 3D FEA. According to most previous FEA studies [34–36] for simulating post-core restored teeth, the experimental results of strength tests showed high correlation with the estimations from the FEA. Since these studies exhibited the possibility and credibility of FEA as an appropriate method for evaluating stresses induced in post and core restored teeth, many studies for evaluating post-core systems have been conducted through FEA [7, 9, 12, 35, 37–39] and this computational simulation method was also used in the present study.

In order to assess the influence of a post-core material on the stress distribution of restored tooth, the magnitudes of VME stresses under functional loading conditions were examined. In all three experimental groups, the highest value of VME occurred at the ceramic crown among the overall components of each restored tooth model. This result is in agreement with the previous study of Sorrentino et al. [37], which conducted an FEA with a tooth model composed of a post, core, and crown, and it revealed that the crown component protects the whole system in the entire post-core restored tooth model.

Regarding the influence of post-core material to the tooth root, the maximum VME stresses were induced at the outer labial region of the root cervical area in all three cases. Comparing the three cases, as the elastic modulus of the post-core decreases, the stress concentration tends to be higher, but the difference is not significant (Figure 3). This result agrees with previous studies [38, 39] that the more rigid the post-core material, the more resistant the deflection forces; therefore, the maximum induced stresses on the root decrease.

Unlike the aforementioned studies, regarding the safety of vertical fracture of post-core treated teeth, other previous studies [7, 9, 12, 35] focused on the importance of the stress distribution profile at the interface of dentine and the post and the induced stress of dentine at the post-apex, rather than the maximum stress concentration at the outer surface of the root. According to the study by Lanza et al. [12], the ideal materials of post-core systems should be sufficiently elastic to accompany the natural flexural movements of the tooth, which more rigid post-core materials cannot do. A post-core material with similar biomechanical properties to dentin could be advantageous in reducing the risk of root fractures. Therefore, a more rigid post-core working against the natural function of the tooth creates zones of tension and shear at the interface of dentine to cemented post. These tensions, the intensities of which depend on the relative rigidity differences between the root and cemented post, can cause debonding or fractures.

Regarding the stress distribution profile in the intraradicular labial root surface, where the interface of dentine to cemented post was located, the PEKK post-core with a similar elastic modulus to dentine showed the lower stress distribution profile along the root mid-line than the higher elastic modulus post-core cases. In the metal and fiberglass cases, the stress distribution profiles were generally higher than for the PEKK case, especially in the cervical and post-apex parts (Figure 4). Taken together, these results showed that the lower elastic modulus post-core material seemed to be favored in the stress distribution profile at the interface region of dentine to cemented post and led to a repairable root fracture mode. These results were similar to those of the aforementioned studies that emphasized the importance of the stress distribution at the interface of dentine and the post.

The fatigue analysis of this FEA study proved that the elastic modulus of post-core material also influences the long-term safety of the overall components in post-core restored teeth. Regarding the mechanical properties of PEKK, its flexural strength was similar to that of dentine and lower than the metal and fiberglass, and it had also a lower elastic modulus than dentine (Tables 1 and 2). As a result, the PEKK induced lower stresses in the post-core component, due to its flexibility, and the FOS value was as high as for other rigid post-core materials. However, when the post material PEKK with lower elastic modulus was used, the stress distribution and safety factor of the post itself were more favored, but rather more stress was transferred to the adjacent cement and crown (Figure 4). As a result, both the post cement and crown in the PEKK post-core model showed relatively low FOS values in the fatigue analysis, which might indicate a higher probability of post cement debonding and crown fracture (Figure 5). In other words, the possibility of vertical fracture of the root is low when post material with low elastic modulus is used, but the possibility of dropping of post-core restoration due to cement debonding can be increased. Therefore, the elastic modulus and strength of the post cement may be influential to the overall system stability in the tooth using a lower elastic modulus post-core material.

In this study, various information was provided regarding the influence of post-core material on biomechanical behavior in an endodontically treated tooth using FEA. However, it is impossible for a computer simulation to include all of the factors in oral environment. Since all structures were assumed as isotropic, homogeneous, linearly elastic, and ideally bonded in this study, direct comparison between the results of this FEA study and clinical outcomes should be made with caution. The properties of tooth structures are not isotropic and homogeneous due to capillary morphologic structure of dentine and prismatic structure of enamel [38]. In addition, under clinical conditions, the post-dentine bonding can be degraded by contamination with water, blood, and saliva [38]. Therefore, further laboratory and clinical studies, including aging effects, are required to verify and supplement the present study.

## 5. Conclusions

Based on the specifications of this study, the following conclusions may be drawn:

1. Although PEKK has a significantly lower elastic modulus and flexural strength than metal and fiberglass, the use of PEKK as a dental post-core system showed potentially high fracture resistance compared to that of metal and fiberglass post-core systems.
2. PEKK post-core, with the lower elastic modulus than dentine, exhibited a favorable stress distribution profile at the intraradicular surface, indicating a lower possibility of root fracture than for conventional post-core materials.
3. Since PEKK transferred higher stresses to the interface material and restorative crown than the other models, due to its flexibility, the probability of debonding and crown failure in PEKK post-core system may be higher than that of rigid post-core systems.

Although the present study is a virtual experiment using 3D FEA, the elastic modulus of post-core material can affect the possibility of vertical fracture of the tooth and post-core debonding. Therefore, understanding the stress distribution and long-term safety of overall post-core restored tooth system would help dental professionals make the right decisions with respect to the choice of post-core material concerning the amount of remaining tooth structure.

## Figures and Tables

**Table 1:** Mechanical properties of the materials used in the finite element model.

Materials	Elastic modulus (GPa)	Poisson's ratio	Reference
Cortical bone	13.7	0.30	[27]
Trabecular bone	1.37	0.30	[27]
Periodontal ligament	0.069	0.45	[27]
Dentine	18.6	0.31	[27]
Gutta-percha	0.69	0.45	[27]
Post cement	5.0	0.30	[27]
Resin core	20.0	0.30	[27]
Fiberglass post	53.8	0.30	[18]
Gold alloy	95.0	0.33	[16]
PEKK	5.1	0.40	Manufacturer
Ceramic crown	62.0	0.30	[27]

**Table 2:** Flexural strengths for the different component materials of the model.

Materials	Flexural strength (MPa)	Reference
Dentine	212.9	[27]
Post cement	97	[27]
Resin core	90	[27]
Fiberglass post	1242.5	[18]
Gold alloy	1545.3	[28]
PEKK	200	Manufacturer
Ceramic crown	160	[27]
