# Corrigendum to “Biomechanical Evaluation of a Tooth Restored with High Performance Polymer PEKK Post-Core System: A 3D Finite Element Analysis”

**DOI:** 10.1155/2017/7196847

**Published:** 2017-11-06

**Authors:** Ki-Sun Lee, Joo-Hee Shin, Jong-Eun Kim, Jee-Hwan Kim, Won-Chang Lee, Sang-Wan Shin, Jeong-Yol Lee

**Affiliations:** ^1^Department of Prosthodontics, Korea University Guro Hospital, Seoul, Republic of Korea; ^2^Department of Conservative Dentistry, Korea University Guro Hospital, Seoul, Republic of Korea; ^3^Department of Prosthodontics, College of Dentistry, Yonsei University, Seoul, Republic of Korea; ^4^Graduate School of Clinical Dentistry, Korea University, Seoul, Republic of Korea

In the article titled “Biomechanical Evaluation of a Tooth Restored with High Performance Polymer PEKK Post-Core System: A 3D Finite Element Analysis” [1], there were some reference and numerical errors in Tables 1 and 2, which should be corrected as follows.

## Figures and Tables

**Table 1:** Mechanical properties of the materials used in the finite element model.

Materials	Elastic modulus (GPa)	Poisson's ratio	Reference
Cortical bone	13.7	0.30	[25]
Trabecular bone	1.37	0.30	[25]
Periodontal ligament	0.069	0.45	[25]
Dentine	18.6	0.31	[25]
Gutta-percha	0.00069	0.45	[25]
Post cement	5.0	0.30	[25]
Resin core	20.0	0.30	[25]
Fiberglass post	53.8	0.30	[13]
Gold alloy	95.0	0.33	[14]
PEKK	5.1	0.40	Manufacturer
Ceramic crown	62.0	0.30	[25]

**Table 2:** Flexural strengths for the different component materials of the model.

Materials	Flexural strength (MPa)	Reference
Dentine	212.9	[25]
Post cement	97	[25]
Resin core	90	[25]
Fiberglass post	1242.5	[13]
Gold alloy	1545.3	[28]
PEKK	200	Manufacturer
Ceramic crown	160	[25]
